# Extraperineal enterocele in male: A case report and literature review

**DOI:** 10.1016/j.ijscr.2020.06.023

**Published:** 2020-06-12

**Authors:** Megumi Asai, Elizabeth Wood, Craig A. Reickert

**Affiliations:** Division of Colon and Rectal Surgery, Henry Ford Hospital, Detroit, MI, United States

**Keywords:** Male enterocele, Extraperineal enterocele, Hedrocele, Rectopexy

## Abstract

**Introduction:**

Enterocele is a herniation of the small bowel through the cul-de-sac. It is uncommon and most often seen in elder females. Large enterocele manifesting as rectal prolapse is exceedingly rare and only few cases are reported previously. Due to it rarity, the best surgical treatment is not yet established especially in male patients. We present a case of enterocele causing rectal prolapse in a male patient that was treated surgically.

**Presentation of case:**

A 47-year-old African American male with chronic constipation and straining presented with manually reducible rectal prolapse. A defecography revealed a large enterocele prolapsing through the anterior rectal wall. The patient underwent an open posterior suture rectopexy with peritoneoplasty. His symptoms completely resolved after surgery, and repeat defecography three months after the procedure showed no sign of recurrence.

**Discussion & Conclusion:**

Extraperineal enterocele in male is a rare disease. Rectopexy with peritoneoplasty can provide a great symptom relieve and improvement on defecography. Long-term outcome should be evaluated.

## Highlights

• Enterocele is uncommon in male patients.
• Enterocele manifesting as rectal prolapse is exceedingly rare, particularly in male.
• Rectopexy with peritoneoplasty is an option for treating large enterocele.

## 1 Introduction

Enterocele is a type of peritoneal hernia involving small bowel protruding through the pouch of Douglas. This is more commonly seen in elderly, multiparous females after a pelvic surgery including hysterectomy [1]. Enterocele is usually associated with other pelvic disorders such as rectal prolapse, rectocele, and cervicocystoptosis [2,3]. Although number of case series and case reports have discussed its pathophysiology, diagnostic and treatment modalities, those literatures predominantly include female patients. Enterocele in male, particularly large symptomatic enterocele, is exceedingly rare. Most male patients lack specific findings on the physical exam and are diagnosed on diagnostic study such as defecography. Due to it rarity, optimum treatment for male enterocele is not well established.

We present a case of a male patient with a large enterocele requiring manual reduction who underwent posterior rectopexy with peritoneoplasty. Our work has been reported in line with the SCARE criteria [4].

## 2 Case

A 47-year-old previously healthy African American male presented to the hospital with chronic constipation and rectal prolapse. He had three-year history of constipation and straining with some mucus drainage via rectum and intermittent hematochezia. His colonoscopy two years prior to his presentation was unremarkable except for mild diverticulosis and internal hemorrhoids. His symptoms continued to progress despite using stool softeners, laxatives and enemas to improve evacuation. He developed rectal pain and rectal prolapse soon after his colonoscopy. His past surgical history includes bilateral inguinal hernia repair. He has no history of trauma or surgery on the pelvic floor.

**Fig. 1:**
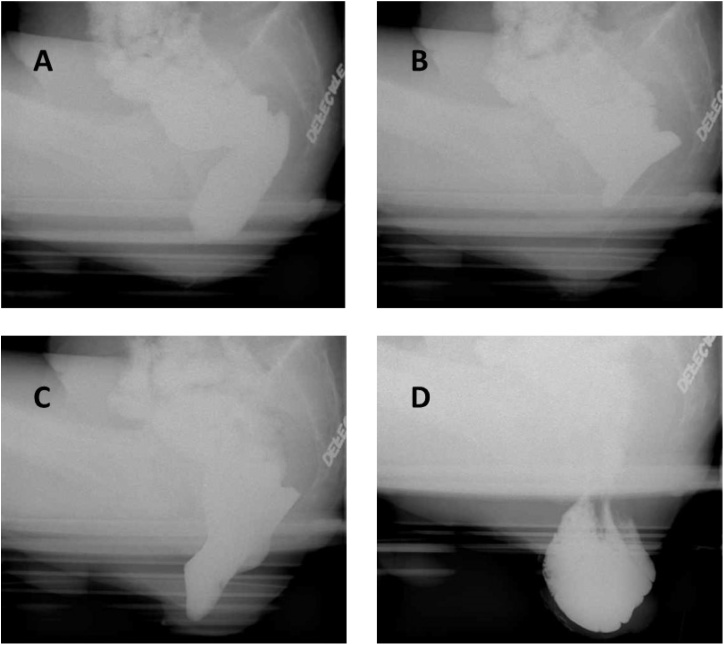
Preoperative defecography. A. At rest with contrast in the small bowel and rectum. B. Normal evacuation of the rectum. C. Small bowel descending to the anus on straining. D. Full thickness rectal prolapse with enterocele.

**Fig. 2:**
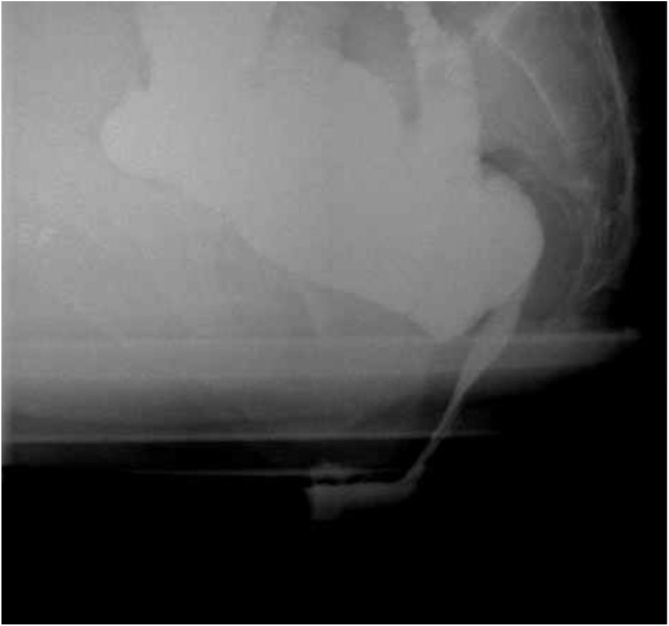
Postoperative defecography. Normal evacuation of the rectum. No recurrence of enterocele.

On examination, he had a 4–5 cm circumferential rectal prolapse on straining which was reducible. A defecography revealed a large enterocele prolapsing behind the prostate and pushing the anterior rectal wall through the anus (Fig. 1). The patient underwent an open posterior rectopexy with peritoneoplasty with a board-certified colon and rectal surgeon. During the surgery, he was found to have broad and deep cul-de-sac and moderately redundant rectum with some posterior laxity in its attachments. The rectum was mobilized posteriorly to the pelvic floor. The rectum was elevated and secured to the sacral promontory with interrupted permanent sutures. Redundant peritoneum anterior to the rectum was then excised and the anterior surface of the rectum was dissected from the anterior structures to the level of the perineal body. The peritoneal edge was closed in two layers with absorbable suture. His postoperative course was unremarkable and he was discharged on the following day. His symptoms were completely resolved after the surgery. Defecography was repeated three months after the procedure and there was no sign of enterocele or rectal prolapse (Fig. 2).

## 3 Discussion

Enterocele was originally reported as a type of vaginal hernia. Sporadic case reports are seen in the 1700’s and 1800’s [5,6]. This was the time before the development of radiologic studies, and diagnosis was made by physical examination and surgical and autopsy findings. Enterocele in the early literatures was divided into vaginal, pudendal and perineal. Small bowel protruding into anterior rectal wall was later reported as hedrocele, and distinguished from enterocele [7,8]. In more recent literatures, enterocele is defined as presence of small intestine in the rectovaginal septum or posterior cul-de-sac regardless of the compression of rectum or vagina [2,3,9]. Therefore, transrectal enterocele and hedrocele are used interchangeably.

Nichols [9] classified the etiology of enterocele as congenital, pulsion, traction and iatrogenic. In previous review, multiparity and pelvic surgery such as hysterectomy are commonly noted in patients with enterocele [[1], [2], [3],[11], [12], [13]]. Enterocele is rare in nulliparous females without preceding surgery [1]. On the other hand, risk factors for male enterocele are not well described. Takahashi et al. [2] reported 18 male patients in their review of total 104 patients with enterocele on defecography. This case series includes one of the largest numbers of male enterocele. However, they lack details of characteristics in this population. Other case series often only include females or identify a very limited number of male patients with enterocele [3,13,14]. Schober et al. [15] and Wester et al. [16] separately reported a similar case of transrectal enterocele in 14-year old male causing obstructing defecation. Both patients had chronic constipation and straining similar to our patient. Chronic abdominal pressure and pulsion may contribute to the development of enterocele with combination of congenital deep cul-de-sac [10].

Symptoms from enterocele varies depends on the size and direction of prolapse. Pelvic discomfort, pelvic pain, obstructing defecation, constipation, fecal incontinence, urinary incontinence, vaginal prolapse are commonly seen [[1], [2], [3],[11], [12], [13]]. Enterocele is often associated with other pelvic organ prolapse and disorder namely cystocele, perineal descent, rectocele, rectal prolapse and rectal intussusception [2,3,11,13]. It is challenging to verify that the symptoms are due to enterocele alone, except for vaginal prolapse or obstructing defecation caused by enterocele confirmed on defecography or pelvic MRI. Transvaginal prolapse could be seen and widening of rectovaginal septum could be felt on bimanual exam in female patients. On rare occasion, visible peristalsis through the vaginal wall may be observed [8]. Physical exam in male patients, on the other hand, is usually unremarkable and enterocele is often diagnosed only after imaging studies.

Large transrectal enterocele manifesting as rectal prolapse is exceedingly rare. Although rectal prolapse is commonly seen with the presence of enterocele, the majority of those patients do not include small bowel in the rectal prolapse. Takahashi et al. [2] reported one extraperitoneal enterocele in rectal prolapse in 61-year old female. Faucheron et al. [17] also described enterocele presenting as full thickness rectal prolapse in 60-year old female. This was the only case in their review of 175 rectal prolapse. Our case, to our best knowledge, is the first case report with extraperitoneal enterocele in male confirmed on defecography.

Although acute complications from transrectal enterocele is uncommon, bowel incarceration is reported [18]. Spontaneous rupture of the rectum and evisceration of small intestine through the anus is another rare condition that may be associated with enterocele. In previous case reports including male and female, most patients had known rectal prolapse before the rupture of the rectum [[19], [20], [21]]. The patients often present with evisceration and are taken to the operating room emergently. It is unclear if the rectal prolapse contained enterocele before it ruptured. However, it is feasible that incarcerated enterocele associated with rectal prolapse can elevate the local transmural pressure and increase the risk of perforation.

The method of enterocele repair is not well established for male patients. In female patients, transvaginal and transabdominal repairs are described [1,[10], [11], [12],^14^]. Some of the techniques used in female including transvaginal repair and sacrocolpopexy are not applied to male patients. In male patients, posterior suture rectopexy with peritoneoplasty, Ripstein rectopexy, peritoneoplasty alone were utilized in previous case reports [2,[14], [15], [16],^18^]. In our case, we elected to use suture rectopexy with peritoneoplasty. The patient had good short-term outcome with resolution of all symptoms of obstructed defecation, rectal prolapse and sensation of rectal fullness.

## 4 Conclusion

Extraperineal enterocele presenting as rectal prolapse is extremely rare, particularly in male patients. Suture rectopexy with perineoplasty can provide symptom relieve and curative treatment. Further follow up is needed to assess long-term outcomes.

## Declaration of competing interest

None.

## Funding

None.

## Ethical approval

Ethical approval was obtained from the Henry Ford Health System Institutional Review Board under category 45 CFR 46.101.

## Consent

Written informed consent was not obtained from the patient. The head of our medical team has taken responsibility that exhaustive attempts have been made to contact the family and that the paper has been sufficiently anonymised not to cause harm to the patient or their family. A copy of a signed document stating this is available for review by the Editor-in-Chief of this journal on request.

## Author contribution

Megumi Asai: conceptualization, investigation, writing – original draft, visualization, Elizabeth Wood: Writing – review & editing, Craig Reickert – conceptualization, resources, supervision.

## Registration of research studies

Not applicable.

## Guarantor

Megumi Asai.

## Provenance and peer review

Not commissioned, externally peer-reviewed.
